# Laser capture microdissection coupled mass spectrometry (LCM-MS) for spatially resolved analysis of formalin-fixed and stained human lung tissues

**DOI:** 10.1186/s12014-020-09287-6

**Published:** 2020-06-17

**Authors:** Jeremy A. Herrera, Venkatesh Mallikarjun, Silvia Rosini, Maria Angeles Montero, Craig Lawless, Stacey Warwood, Ronan O’Cualain, David Knight, Martin A. Schwartz, Joe Swift

**Affiliations:** 1 The Wellcome Centre for Cell-Matrix Research, University of Manchester, Manchester, M13 9PT UK; 2 Division of Cell Matrix Biology and Regenerative Medicine, School of Biological Sciences, Faculty of Biology, Medicine and Health, University of Manchester, Manchester Academic Health Science Centre, Manchester, M13 9PL UK; 3 Histopathology Department, Manchester University NHS Foundation Trust, Southmoor Road, Wythenshawe, Manchester, M23 9LT UK

## Abstract

**Background:**

Haematoxylin and eosin (H&E)—which respectively stain nuclei blue and other cellular and stromal material pink—are routinely used for clinical diagnosis based on the identification of morphological features. A richer characterization can be achieved by laser capture microdissection coupled to mass spectrometry (LCM-MS), giving an unbiased assay of the proteins that make up the tissue. However, the process of fixing and H&E staining of tissues provides challenges with standard sample preparation methods for mass spectrometry, resulting in low protein yield. Here we describe a microproteomics technique to analyse H&E-stained, formalin-fixed paraffin-embedded (FFPE) tissues.

**Methods:**

Herein, we utilize heat extraction, physical disruption, and in column digestion for the analysis of H&E stained FFPE tissues. Micro-dissected morphologically normal human lung alveoli (0.082 mm^3^) and human lung blood vessels (0.094 mm^3^) from FFPE-fixed H&E-stained sections from Idiopathic Pulmonary Fibrosis (IPF) specimens (n = 3 IPF specimens) were then subject to a qualitative and then quantitative proteomics approach using BayesENproteomics. In addition, we tested the sensitivity of this method by processing and analysing a range of micro-dissected human lung blood vessel tissue volumes.

**Results:**

This approach yields 1252 uniquely expressed proteins (at a protein identification threshold of 3 unique peptides) with 892 differentially expressed proteins between these regions. In accord with prior knowledge, our methodology approach confirms that human lung blood vessels are enriched with smoothelin, CNN1, ITGA7, MYH11, TAGLN, and PTGIS; whereas morphologically normal human lung alveoli are enriched with cytokeratin-7, -8, -18, -19, 14, and -17. In addition, we identify a total of 137 extracellular matrix (ECM) proteins and immunohistologically validate that laminin subunit beta-1 localizes to morphologically normal human lung alveoli and tenascin localizes to human lung blood vessels. Lastly, we show that this micro-proteomics technique can be applied to tissue volumes as low as 0.0125 mm^3^.

**Conclusion:**

Herein we show that our multistep sample preparation methodology of LCM-MS can identify distinct, characteristic proteomic compositions of anatomical features within complex fixed and stained tissues.

## Background

Mass spectrometry (MS) proteomics is a powerful tool to systemically identify and quantify proteins in complex biological samples. The utility of this method is maximized when performed with spatial resolution to report on the composition and function of specific regions of tissue. Extracellular matrix (ECM) is particularly important in determining cell behaviour in health and disease [1] but is especially challenging for proteomic analysis given the extensive covalent crosslinking and low solubility of many ECM proteins [2]. However, common protocols for bottom-up proteomics (i.e. based on detection of peptide protein fragments) require sample homogenization and digestion, resulting in a loss of any information regarding protein localization and spatial relationships. To this end, laser capture microdissection coupled to mass spectrometry (LCM-MS) is a method currently being optimized for microproteomics to determine regional tissue differences [3]. LCM-MS has been performed using fresh [4], flash-frozen [5–7], and formalin-fixed paraffin-embedded (FFPE) tissues [8–11]. In this study, we describe and examine the performance of a protocol for LCM-MS analysis of FFPE sections of human lung tissue that were haematoxylin and eosin (H&E) stained.

H&E staining provides critical morphological characterization enabling researchers to identify anatomical features of interest. However, haematoxylin staining has been shown to reduce protein detection by MS [12]. Using H&E-stained frozen tissue (not FFPE), groups have been able to develop highly-sensitive microproteomic techniques to detect 1500 to 1824 unique proteins from laser capture microdissected brain tissues with volumes as low as 3.8–6.0 × 10^−4^ mm^3^ [13, 14]. A limitation to translational studies is that clinical specimens are widely stored as FFPE tissue blocks. As a result, few LCM-MS studies exist for H&E-stained FFPE tissue sections. In an earlier study, up to 866 proteins [15] were identified from H&E stained FFPE sections of human head and neck squamous cell carcinomas; and up to 714 unique proteins from cutaneous squamous cell carcinoma H&E stained FFPE tissue [16]. This sensitivity gap supports the need for novel LCM-MS protocols for H&E stained FFPE tissues.

Herein, we describe and demonstrate the application of a protocol for microproteomics that combines multiple steps that have been shown to individually enhance protein yield. First, we perform a detergent-based heat-retrieval procedure which enhances protein solubility by reversing chemical crosslinking caused by fixation [17–19]. We combined this with two techniques to enhance extraction of extracellular matrix (ECM) proteins: physical disruption [10, 20] and chemical extraction with a urea-based buffer [21]. These steps reflect the importance of ECM as modulators of fibrosis and cancer [22, 23]. Lastly, we utilize an in-column trypsin-digest system (a recently commercialized product, SuspensionTrap [24, 25]) shown to increase peptide yields [12] while effectively removing detergents and contaminants from the samples [26]. Our data analysis suggests that our protocol may delineate between cellular and ECM protein components characteristic of the different regions of FFPE H&E-stained tissue.

## Materials and methods

### Procurement of human lung tissue

The use of human lung tissue was approved by University of Manchester Health Research Authority with patient consent under protocol REC#14/NW/0260. The specimens used for this study met the criteria for Idiopathic Pulmonary Fibrosis (IPF) diagnosis [27], however, we used distal lung tissue that appeared morphologically normal for the LCM-MS study.

### Immunohistochemistry

Human lung samples were formalin-fixed and paraffin-embedded (FFPE). Deparaffinized and rehydrated 5-micron sections were subjected to antigen heat retrieval using citrate buffer (Abcam, ab208572), for 30 min at 100 °C, cooled to room temperature for 20 min, treated with 3% hydrogen peroxide for 5 min, blocked in TBS Super Block for 1 h (Thermo Fisher; 37581), and probed with primary antibody (TNC, 1:500, Abcam, ab108930; LAMB1, 1:1000, Abcam, ab16048) overnight in 10% blocking solution. The following day, the specimens were subjected to Novolink Polymer Detection Systems (Leica RE7270-RE, per the manufacturer’s recommendations), developed for 5 min with DAB Chromagen (Cell Signal, 11724), counterstained with haematoxylin and cover-slipped with Permount (Thermo Fisher Scientific, SP15).

### Pentachrome staining

We followed a modified Russell-Movats pentachrome staining protocol. Deparaffinized specimens were stained with alcian blue for 20 min (1% alcian blue [Sigma-Aldrich, A-1986] and 1% glacial acetic acid), treated in alkaline alcohol for 1 h (90% alcohol and 10% of a 30% ammonium hydroxide solution [Sigma-Aldrich, 221228]), alcohol haematoxylin solution for 10 min (50% of a 5% absolute alcoholic haematoxylin [Sigma Aldrich, P4006], 25% of a 10% aqueous ferric chloride [Sigma-Aldrich, F2877], and 25% of 2 grams Iodine [Alfa Aesar, A12278], 4 grams potassium iodide [Fluorochem, 319032] in 100 mL water), 5% aqueous sodium thiosulfate for 1 min (Sigma-Aldrich, S-6672), and then crocein scarlet-acid fuchsin solution for 2 min (4 parts crocein scarlet—0.1% crocein scarlet [Alfa Aesar, J66876] and 0.5% glacial acetic acid; 1 part acid fuchsin—0.1% acid fuchsin [Alfa Aesar, B22222] and 0.5% glacial acetic acid). Sections were then treated 2 times with 5% phosphotungstic acid for 5 min each (Sigma Aldrich, P4006), washed 3 times with 100% alcohol, stained with 6% alcoholic saffron (VWR, 283-295-0) for 15 min, and then coverslipped with Permount.

### Haematoxylin and eosin staining

5-micron FFPE sections were mounted onto MMI membrane slides (MMI, 50102) and stained using an automated stainer (Leica XL) at the Histology Core at University of Manchester. In short, the FFPE slides were dewaxed by xylene and alcohol treatment, followed by a 2-minute hematoxylin incubation, acid alcohol treated, and stained with eosin for 1 min. Slides were then washed in 100% ethanol and allowed to air dry. Slides were then stored in 4 °C for up to 1 week before LCM-MS.

### Histological imaging

Stained slides were imaged using a DMC2900 Leica camera along with Lecia Application Suite X software (Leica).

### Laser capture microdissection

The 5-micron H&E slides were loaded onto the MMI CellCut Laser Microdissection system (Molecular Machines & Industries). Using MMI CellCut software, we performed a complete slide scan using a CellScan toolbar at 4× magnification to make navigation easier. Using a computer mouse, we used a closed-shape manual drawing tool to select our region of interest. We then focused our laser at 350 μm and performed an automated cutting using a 60% laser power setting moving at 50 μm/sec. To collect microdissected specimens, we used adhesive MMI transparent caps (MMI, 50204) and MMIs CapLift technology to gently lift and store specimens onto the adhesive caps. Captured specimens were stored at − 20 °C for several weeks until all samples were collected and processed for mass spectrometry.

### Sample preparation for mass spectrometry

Laser microdissected tissue was resuspended in 25 μL 50 mM triethylammonium bicarbonate (TEAB) (Sigma, T7408), 5% SDS (pH 7.5) and subjected to 95 °C for 20 min, then 60 °C for 2 h while shaking at 1400 RPM (Eppendorf, ThermoMix C). We then added 75 μL of a 50 mM TEAB, 5% SDS, 10 M urea, 13.3 mM DTT (pH 7.5) solution to the 25 μL sample, after it had cooled to room temperature to avoid deamination, to create a final volume of 100 μL of 50 mM TEAB, 5% SDS, 7.5 M urea, 10 mT DTT (pH 7.5). Samples were then placed into a Covaris microtube (Covaris, 520045) and sheared using the LE220-Plus Focused Ultrasonicator (Covaris, UK) set at 6 °C with the following settings: duration of 50 s, peak power 500, duty factor of 20.0%, cycles/burst of 200, average power at 100, and then delayed for 10 s. This was repeated for a total of 10 cycles (10-min total run time). The homogenization process resulted in the break-up of bulk pieces of tissue, leaving only a fine suspension of material. A benefit of the LE220-Plus Focus Ultrasonicator is that it allows for the processing of up to 96 samples in parallel in a single run. After shearing, samples were alkylated by the addition of 8 µL of 500 mM iodoacetamide (Sigma, I1149) and incubated for 30 min in the dark. Samples were then acidified by the addition of 12 μL of 12% aqueous phosphoric acid (Sigma, 345245) and centrifuged at 12000 RPM for 5 min. The supernatant was collected and resuspended with 600 µL of 90% methanol, 100 mM TEAB (pH 7.10). The sample was then added to a micro S-Trap column (ProtiFi, C02-micro) and centrifuged at 2000 RPM using 200 µL at a time until all the sample had passed through the column. After discarding the flow through, the S-Trap column was washed by adding 150 μL of 90% methanol, 100 mM TEAB (pH 7.10) and centrifuging at 2000 RPM. Washing was repeated a further 9 times, discarding the flow through each time. In-column digest was performed by adding 25 μL of a 0.8 µg/µL trypsin solution (proteomics grade trypsin; Promega, V5111) in 50 mM TEAB pH 8.0 in accordance with the manufacturer’s protocol for 1-hour digestion. Trypsin digestion was performed at 47 °C for 1 h without shaking. Samples were eluted by adding 40 μL of 50 mM TEAB (pH 8.0) and centrifuging at 2000 RPM, followed by the addition of 40 μL of 0.2% aqueous formic acid (Sigma Aldrich, 27001) and centrifuging, and finally adding 40 μL 50% aqueous acetonitrile (Fisher, A955-212) and centrifuging. Eluted fractions were combined and the total 120 μL sample was then lyophilised using a speed-vac (Heto Cooling System).

Desalting of samples was performed using Oligo R3 resin beads. Briefly, 100 µL of a 10 mg/mL settled Oligo R3 resin (Thermo Scientific, 1-1339-03) in aqueous 50% acetonitrile was placed into a 96-well 0.2 µm PVDF filter plate (Corning, 3504). The plate was centrifuged at 1400 RPM (Thermo Scientific, Megafuge 16) for 1 min to clear Oligo R3 resin with a blank 96-well plate underneath to catch the flow through which was then discarded. 100 μL of aqueous 50% acetonitrile was mixed with the resin and centrifuged again, discarding the flow through. Finally, 100 μL of aqueous 0.1% formic acid were mixed with the resin and centrifuged for a total of two repeats, while discarding the flow through. Samples were then resuspended in 100 µl of aqueous 5% acetonitrile, 0.1% formic and mixed with the now washed Oligo R3 Resin and allowed to shake on a plate shaker (Eppendorf, Thermomixer Comfort) for 5 min at 800 RPM, and then centrifuged (flow through was discarded). The sample peptides were now bound to the Oligo R3 Resin and washed for a total of ten times by the addition of 100 µL of aqueous 0.1% formic acid, mixed for 2 min at 800 RPM, centrifuged, and flow through discarded. Finally, the washed peptides were eluted by mixing with 50 µL of aqueous 50% acetonitrile for 2 min at 800 RPM, centrifuged, collecting the flow through in a clean 96-well capture plate. Elution was repeated with an additional 50 μL of aqueous 50% acetonitrile and retained. Desalted peptides were lyophilized in a speed-vac and stored at 4 °C until needed.

### Liquid chromatography coupled tandem mass spectrometry

Lyophilized peptides were resuspended in 10 μL of a 5% acetonitrile, 0.1% formic acid solution and evaluated by liquid chromatography (LC) coupled tandem MS (LC-MS/MS) using an UltiMate^®^ 3000 Rapid Separation LC system (RSLC, Dionex Corporation, Sunnyvale, CA) coupled to a Q Exactive HF mass spectrometer (Thermo Fisher). To maximize the sensitivity of the system, it was configured to directly inject onto the analytical column (temperature set at 35 °C) without a trap. Mobile phase A was 0.1% formic acid in water and mobile phase B was 0.1% formic acid in acetonitrile and the column used was a 75 mm × 250 μm i.d. 1.7 µM CSH C18, analytical column (Waters). The analytical method used was as follows: a 1 μL aliquot of the sample (i.e. 10% of the total peptides) was transferred to a 5 μL injection loop to increase the amount of sample analyzed at a flow rate of 300 nL/min for 5 min at 5% B. The loop was then taken out of line and the peptides were separated using a gradient that went from 5% to 7% B at 200 nL/min in 1 min, followed by a shallow gradient from 7% to 18% B in 64 min, then from 18% to 27% B in 8 min, and finally from 27% to 60% B in minute. At 85 min, the flow is increased to 300 nL/min until the end of the run at 90 min.

Mass spectrometry data was acquired in a data directed manner for 90 min in positive mode. Peptides were selected for fragmentation automatically by data dependent analysis on a basis of the top 12 peptides with m/z between 300 and 1750Th and a charge state of 2, 3 or 4 with a dynamic exclusion set at 15 s. The MS Resolution was set at 120,000 with an automatic gain control (AGC) target of 3e6 and a maximum fill time set at 20 ms. The MS2 Resolution was set to 30,000, with an AGC target of 2e5, a maximum fill time of 45 ms, isolation window of 1.3Th and a collision energy of 28.

### Mass spectrometry data analysis and statistics

Raw spectra were automatically aligned using Progenesis QI for proteomics (version 4.1; Nonlinear Dynamics, Waters). Progenesis QI’s alignment feature allows MS2 spectral information to be shared across samples so that MS1 spectra that do not have associated MS2 spectra in a given sample (owing to low abundance and thus not being selected for fragmentation in data-dependent acquisition) can still be identified. Alignment decreases the number of missing values (a common problem in proteomics) at the risk of wrongly inferring presence of a peptide when it may genuinely be absent. Spectra from different tissue sections (human lung blood vessel or morphologically normal human lung alveoli) were analysed either separately (with alignment between donors but not between sections; Fig. 2) or together (with alignment between both sections and donors; Fig. 3). Peak-picking in Progenesis QI was performed using default parameters which include charges from + 1 to + 4 (an inclusion useful for downstream troubleshooting) and subsequent features were then filtered to leave only peptides with a charge of + 2 and + 3, with 3 or more isotopes. Remaining features were searched using Mascot (server version 2.5.1, parser version 2.5.2.0; Matrix Science), against the SwissProt and TREMBL human database. The peptide database was modified to search for alkylated cysteine residues (monoisotopic mass change, +57.021 Da) as a fixed modification, with oxidized methionine (+ 15.995 Da), hydroxylation of asparagine, aspartic acid, proline or lysine (+ 15.995 Da) and phosphorylation of serine, tyrosine, threonine (+ 79.966 Da) as variable modifications. A maximum of two missed cleavages was allowed. Peptide tolerance and MS/MS tolerance were set to 8 ppm and 0.015 Da, respectively. Peptide detection intensities were exported from Progenesis QI as comma separated variable (.csv) spreadsheets for further processing. Peptides assigned to proteins with ‘unreviewed’ status in the UniProt database were reassigned to the most abundant ‘reviewed’ protein with sequence identity in the dataset. Peptides shared between different protein groups were excluded from subsequent analysis. For differential expression analysis in Fig. 3, fold changes were calculated using the Matlab (version 2015a; The MathWorks) implementation of BayesENproteomics [28], available from: https://github.com/VenkMallikarjun/BayesENproteomics. BayesENproteomics fits regularized regression models to take into account donor variability and variable behaviour of peptides assigned to a single protein group (due to post-translational modifications or differential splicing), weighting observations based on confidence in peptide identification (inferred via Mascot scores). These features allow BayesENproteomics to calculate fold changes for the dominant proteoforms of proteins represented in a complex clinical dataset. Missing values were imputed within BayesENproteomics model fitting using an adaptive multiple imputation method that attempts to discern whether a given missing value is missing at random (MAR) or non-randomly (MNR) and imputes from appropriate distributions [28]. Reactome [29, 30] Pathway enrichment analysis was performed as described in [28], by fitting linear models for each pathway represented in the dataset, based on protein-level fold changes calculated as described above.

### Tissue titration data analysis

Samples were loaded to the MS as described above. To restrict carryover of the previous sample, we loaded the MS with the lowest tissue volume sample first followed by increasing tissue volume samples. Raw spectra were processed using MaxQuant (version 1.6.10.43) [31] against the human proteome obtained from Uniprot (March 2020) [32]. Default settings were used with variable modifications as methionine oxidation and N-terminal acetylation, fixed modification of carbamidomethylation of cysteine and match between runs was selected to allow MS/MS identifications to be transferred across samples.

### Data availability

Raw mass spectrometry data were deposited to ProteomeXchange with the identifier PXD014762.

## Results

### Microproteomics for haematoxylin & eosin-stained formalin-fixed paraffin-embedded tissue

**Fig. 1:**
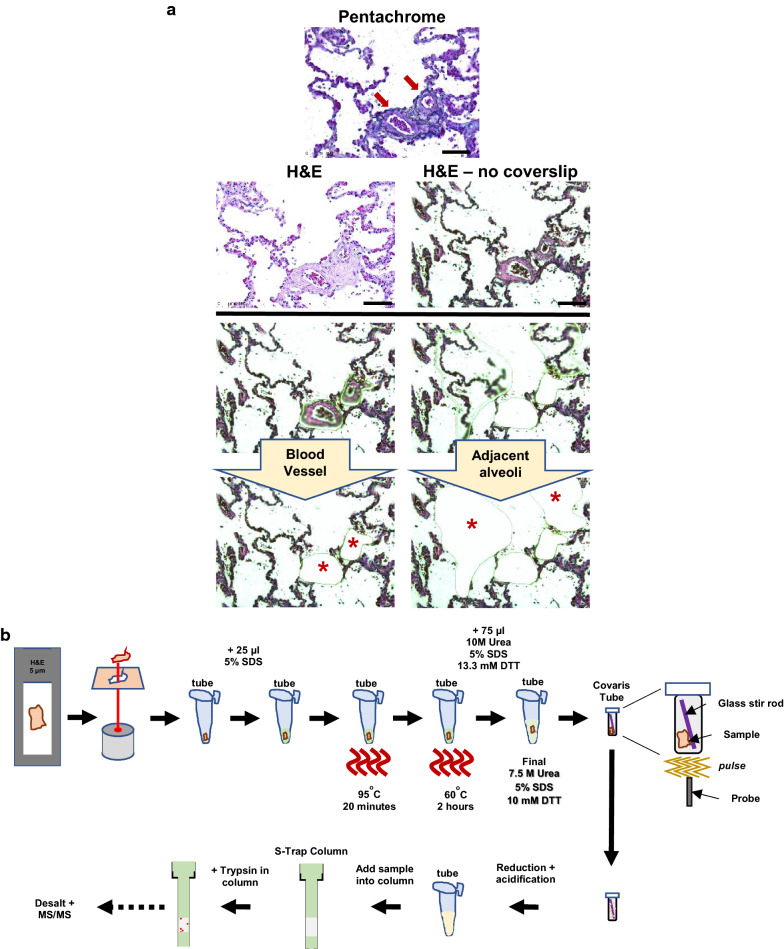
Laser capture microscopy of human lung blood vessels for mass spectrometry. **a** Blood vessels are identified by pentachrome stain (red arrows) and serial sections are used to laser microdissect blood vessels and adjacent alveoli. **b** A workflow of tissue preparation for mass spectrometry. Laser captured tissue is detergent treated, subjected to heat, resuspended with Urea, sheared using Covaris, and samples are later placed into a S-Trap Column for trypsin digest, followed by desalting prior to mass spectrometry loading

Morphologically normal human lung alveoli and human lung blood vessels were laser microdissected from uninvolved IPF tissue (Fig. 1a) using a pentachrome and H&E stain as a guide. Per specimen (n = 3 IPF patients), approximately 0.082 mm^3^ of morphologically normal human lung alveoli and 0.094 mm^3^ of human lung blood vessels were pooled from H&E-stained 5-micron FFPE sections and used for downstream mass spectrometry; encompassing a total of 6 LCM-MS samples (Schematic in Fig. 1b). In short, samples were resuspended in 5% SDS, and heated to denature proteins, then resuspended at room temperature to a final solution of 7.5 M urea, 5% SDS, and 10 mM DTT to enhance ECM solubility [21]. Samples were then sheared using a LE220 focused-ultrasonicator; a 96-multiwell system which allows for scalability (Covaris Ltd, United Kingdom). This combined protocol extracted more proteins as assessed by SDS-PAGE than individual methods (Additional file 1: Figure. S1). Samples were then placed into an S-Trap column (ProtiFi, NY, USA) [25], desalted, and analysed for mass spectrometry.

### The ECM comprising morphologically normal human lung alveoli and blood vessels

**Fig. 2:**
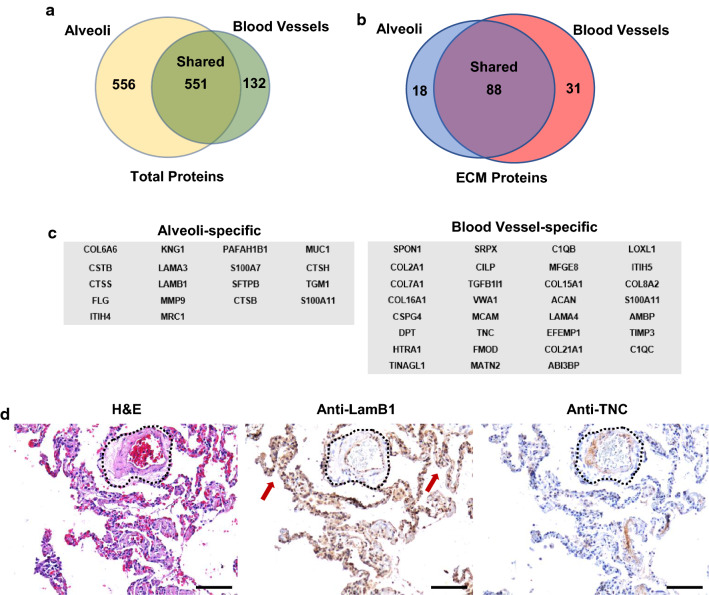
The ECM comprising morphologically normal alveoli and blood vessels in IPF. **a**–**b** A Venn diagram showing the number of (**a**) total and (**b**) ECM proteins within morphologically normal alveoli and blood vessels (n = 3 IPF specimens). **c** A list of ECM genes specific for morphologically normal alveoli and blood vessels. **d** Serial sections stained with H&E, anti-LamB1 and anti-TNC. A blood vessel is outlined in black dots, red arrows highlight intense immunostain for LamB1, and black arrow highlights immunostain for TNC within the blood vessel. Scale bar represents 100 μm

We first performed a qualitative analysis of our data using Progenesis QI (Nonlinear Dynamics) and Mascot (Matrix Science). With a cut-off of 3 or more peptides, we identified 1107 and 683 proteins in morphologically normal human lung alveoli and human lung blood vessels, respectively (Fig. 2a). We then used the Human Matrisome Project (http://matrisomeproject.mit.edu) which provides a list of core matrisome proteins (ECM glycoproteins, collagens, and proteoglycans) and matrisome-associated proteins (ECM-affiliated proteins, ECM regulators, and secreted factors) and directly compared to our protein list [33]. This analysis identified 106 and 119 ECM proteins in morphologically normal human lung alveoli and human lung blood vessels, respectively. We show that 88 ECM constituents are shared within these tissues (Additional file 2: Table S1), with 18 proteins unique to morphologically normal human lung alveoli and 31 proteins unique to human lung blood vessels (Fig. 2b). This approach utilizes multi-steps to enrich for ECM proteins and we report that 11.1% of the proteins detected are ECM components. This is in accord with studies utilizing human lung specimens where the percentage of ECM components detected are reported as 5.4% [34], 7.2% [35], and 8.2% [36]; in mouse lungs, 5.2% is reported [37]. The protein gene names for these regions are shown in Fig. 2c. To validate these results, we carried out immunostaining for two proteins that proteomics identified as specific to each tissue. Antibody to laminin subunit beta-1 (LAMB1) stained predominantly the alveoli (red arrows), whereas antibody to tenascin (TNC) stained blood vessels but not alveoli (blood vessel outlined in black dots; Fig. 2d).

**Fig. 3:**
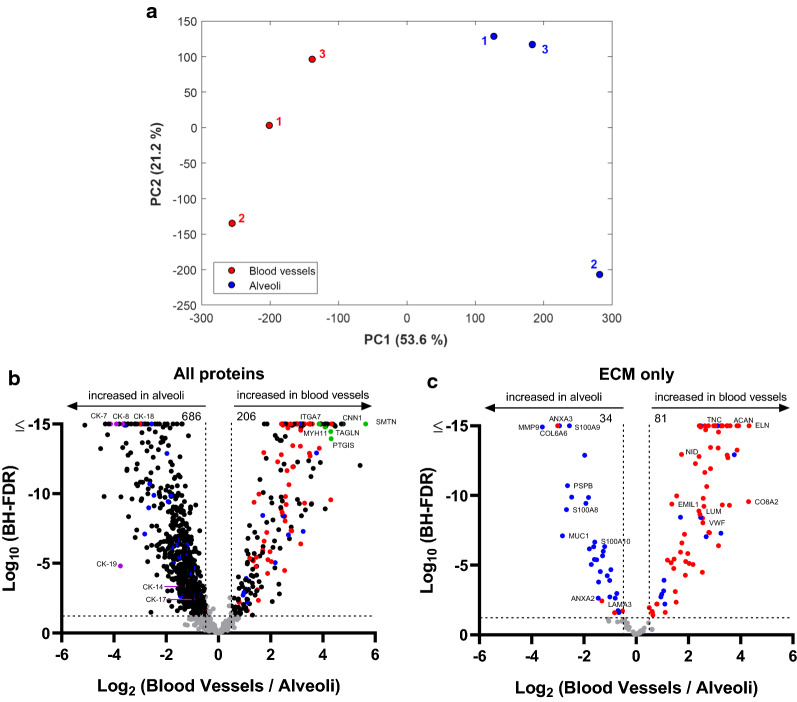
The ECM comprising morphologically normal alveoli and blood vessels in IPF. **a** Principal component analysis (PCA) of normalised peptide intensities for the micro-dissected human lung blood vessels (red) and alveoli (blue). Numbers next to dots denote donor IDs. Values in brackets denote percentage of variance explained by each principal component (PC). **b** A volcano plot of all 1252 proteins or (**c**) ECM proteins only showing a negative natural log of the FDR values plotted against the base 2 log of the change for each protein. The thresholds are set for a base log 2 > 0.5 and FDR *p* value < 0.05; n = 3 IPF specimens. Purple dots indicate known proteins expressed in alveoli, green dots indicate known proteins expressed in blood vessels, blue dots represent matrisome-associated proteins, and red dots represent core-matrisome proteins

We next used a quantitative approach to gain further insight into the differences in the total protein abundance between morphologically normal human lung alveoli and human lung blood vessel. This was done by utilizing Progenesis QI to align all samples from different sections together, followed by BayesENproteomics [28] to compare the differential expression of the 1252 proteins identified (at a protein identification threshold of 3 unique peptides). Principal component analysis (PCA) on normalized peptide intensities prior to protein quantification showed that sample handling and data processing did not perturb the relationship between samples from different tissue sections (Fig. 3a). Subsequent analysis of protein fold changes showed that 206 proteins are enriched in blood vessels whereas 686 are enriched in morphologically normal alveoli (Additional file 3 Fig. 3b). In accordance with prior knowledge, proteins known to be expressed in blood vessels, such as smoothelin, CNN1, ITGA7, MYH11, TAGLN, and PTGIS are over-represented in the blood vessels (highlighted in green dots) [38–41]. The top 10 pathways and top 10 proteins enriched in human lung blood vessels are shown in Tables 1 and 2, respectively. Similarly, cytokeratin-7, -8, -18, -19, 14, and -17 are enriched in the alveoli as previously shown (highlighted in purple dots) [42, 43]. The top 10 pathways and top 10 proteins enriched in alveoli are shown in Tables 3 and 4, respectively.

**Table 1:** Reaction pathways enriched in blood vessels

Pathway name/reactome pathway identifier (R-HSA)	Effect size	FDR	Set size
Collagen chain trimerization/R-HSA-8948216	2.71	1.83E−14	22
ECM proteoglycans/R-HSA-3000178	2.20	2.12E−14	36
Collagen biosynthesis and modifying enzymes/R-HSA-1650814	2.23	2.22E−11	26
Non-integrin membrane-ECM interactions/R-HSA-300171	2.36	9.69E−11	20
Molecules associated with elastic fibres/R-HSA-2129379	2.21	1.11E−10	15
Syndecan interactions/R-HSA-3000170	2.45	1.11E−10	12
Extracellular matrix organization/R-HSA-1474244	2.84	3.42E−10	10
MET activates PTK2 signaling/R-HSA-8874081	2.26	9.02E−10	15
Collagen degradation/R-HSA-1442490	2.18	3.12E−09	16
Laminin interactions/R-HSA-3000157	1.95	2.36E−08	6

**Table 2:** Proteins enriched in blood vessels

Gene symbol; name	Log2 fold-change	FDR
SMTN; smoothelin	5.63	1E−15
MYPT2; myosin phosphatase-targeting subunit 2	5.42	9.23E−13
DMD; dystrophin-1	4.78	1E−15
CNN1; calponin-1	4.73	1E−15
LMOD1; leiomodin-1	4.70	1E−15
SYUG; gamma-synuclein	4.47	1.44E−15
MYL9; myosin RLC	4.44	1.22E−15
FLNC; filamin-C	4.38	1E−15
ELN; elastin	4.32	1E−15
PTGIS; prostacyclin synthase	4.31	1.12E−14

**Table 3:** Reactome pathways enriched in alveoli

Pathway name/reactome pathway identifier (R-HSA)	Effect size	FDR	Set size
Neutrophil degranulation/R-HSA-6798695	1.06	1.83E−14	162
Regulation of expression of SLITs and ROBOs/R-HSA-9010553	0.99	1.83E−14	283
L13a-mediated translational silencing of Ceruloplasmin expression/R-HSA-156827	1.19	1.83E−14	61
Translation initiation complex formation/R-HSA-72649	1.21	1.83E−14	33
Formation of a pool of free 40S subunits/R-HSA-72689	1.20	1.83E−14	55
Formation of the ternary complex, and subsequently, the 43S complex/-HSA-72695	1.22	1.83E−14	29
Ribosomal scanning and start codon recognition/R-HSA-72702	1.20	1.83E−14	34
GTP hydrolysis and joining of the 60S ribosomal subunit/R-HSA-72706	1.18	1.83E−14	62
mRNA splicing—major pathway/R-HSA-72163	1.23	1.83E−14	42
SRP-dependent cotranslational protein targeting to membrane/R-HSA-1799339	1.23	1.83E−14	56

**Table 4:** Proteins enriched in alveoli

Gene symbol; name	Log2 fold-change	FDR
CAH4; carbonic anhydrase	5.11	1.22E−15
ACSL5; long-chain-fatty-acid CoA ligase 5	4.50	6.11E−13
AMPE; aminopeptidase A	4.37	1.04E−09
AQP4; aquaporin-4	4.32	1.65E−14
RAGE; advanced glycosylation end product-specific receptor	4.24	7.52E−14
ABCA3; ATP-binding cassette sub-family A member 3	4.19	1.65E−14
K2C7; cytokeratin-7	4.12	1.65E−14
K1C18; cytokeratin-18	3.90	1.65E−14
PCAT1; LPC acyltransferase 1	3.80	1.65E−14
K1C19; cytokeratin-19	3.75	4.17E−05

We next specifically examined the core-matrisome (red dots) and matrisome-associated (blue dots) proteins (Fig. 3c). Consistent with prior knowledge, blood vessels have been shown to be enriched in aggrecan (ACAN), elastin (ELN), emilin (EMIL1), lumican (LUM), tenascin (TNC), von Willebrand factor (VWF), nidogen (NID1), and collagen VIII (CO8A2) which are shown in the volcano plot [44]. Similarly, ECM proteins MMP9, MUC1, PSPB (surfactant protein B), S100A8/A9, ANXA2, and collagen VI are all known to be enriched in lung epithelium in the context of IPF pathogenesis which are shown in the volcano plot [45–50]. Together, these data further support that our microproteomics protocol, using multiple techniques to process samples, may be useful to delineate the ECM composition between distinct regions.

### Titration of hematoxylin & eosin-stained formalin-fixed paraffin-embedded tissue for microproteomics

**Fig. 4:**
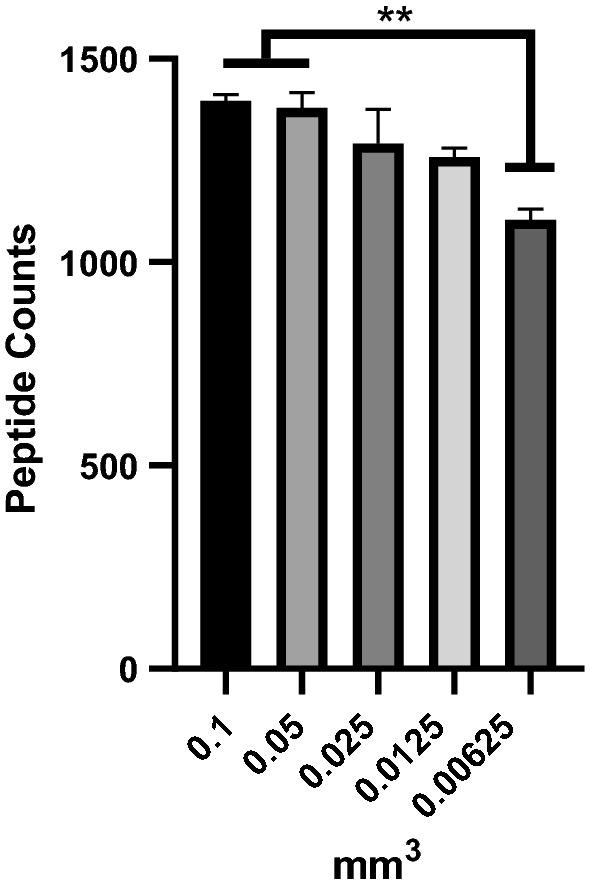
Peptide counts of varying volumes of laser capture microdissected tissue subjected to mass spectrometry. Blood vessels from one IPF specimen was laser capture microdissected at given volumes and prepared for mass spectrometry with peptide counts shown (n = 1, with each condition performed in 3 technical replicates); ** p < 0.01 (one-way ANOVA with Tukey post hoc testing)

We next sought to determine the sensitivity of our LCM-MS protocol by titrating varying volumes of H&E-stained FFPE human lung tissue. We chose to laser capture microdissect human lung blood vessels at a volume of 0.1, 0.05, 0.025, 0.0125, and 0.00625 mm^3^. Using Maxquant to process our data, we plot the volume of tissue versus number of peptide counts (using 2 unique peptides) (Fig. 4). Herein, we find that peptide counts are statistically decreased at our lowest volume of 0.00625 mm^3^ of FFPE H&E stained tissue, and we see a modest decrease in peptide counts with decreasing volume input.

## Discussion

The methods described use techniques to insure sensitivity and reproducibility. The Covaris focused ultrasonication system provided a highly efficient method of sample disruption with a format that allows up to 96-samples to be processed as a batch with both the amount of energy applied and temperature controlled precisely. The S-Trap digestion method was performed in parallel and we found that it provided efficient sample recovery as previously reported [24, 25]. To desalt samples, we used POROS R3 beads as they are highly hydrophobic which retain hydrophilic peptides, and have a low susceptibility for clogging [51]. We found that incubating the beads during extraction increases recovery over tip based methods as the time for adsorption on to the beads are long and more regulated. The plate based format allows for samples to be rapidly cleaned in parallel. In addition, the liquid chromatography system was adjusted for sensitivity and speed. To increase sensitivity, the system is configured for direct injection on to the analytical column (i.e. no trap column is used) with low flowrates of 200 nl/min; In our experiences we find that this supports chromatographic resolution, peak capacity, signal intensity, and decreases sample complexity. To increase speed, our experience show that using low injection volumes and a high loading rate of 300 nL/min supports signal intensity without impacting chromatograph resolution. To insure minimal dead volume and peak broadening, junctions after the column were made butt-to-butt with polished capillaries. Taken together, we found that these features improved our MS methodology.

Common proteomics-based efforts to map tissue composition are limited by the loss of spatial information caused by the need to completely homogenize tissue pieces during sample preparation. Here we describe a micro-proteomics strategy using tissue processed by the most widely used staining technique to identify regions of interest, followed by microdissection and subsequent proteomics. With the limitation of a small sample size (n = 3), we determined the ECM composition of morphologically normal lung alveolar structures as compared to adjacent human lung blood vessels in unaffected regions from IPF lungs. To address the many challenges including low tissue volumes (< 0.1 mm^3^), formalin-fixation, and histological stains, we utilized a variety of strategies employing commercially available tools to qualitatively show a yield of 1107 and 683 unique proteins to morphologically normal human lung alveoli and human lung blood vessels, respectively, and identified a total of 137 as ECM proteins. Using BayesENproteomics as a quantitative approach [28], we identify 1252 unique proteins with 892 differentially expressed proteins between these regions using a strict threshold of 3-unique peptides. Using 2 unique peptides as the threshold, we would identify a total of 1737 unique proteins using this approach (485 more proteins).

One rate limiting step to this approach is the time required to micro-dissect tissue. In this study, it took about 15 h of laser capture microscope time to capture both human lung blood vessels and morphologically normal lung alveoli at roughly 0.1 mm^3^, per patient. This would become a challenge if a region of interest is a small cell cluster which would require many sections, slides, and other resources to accomplish. It was therefore imperative to determine the sensitivity of our approach by titration of starting volumes and then subjecting the material micro-proteomics. Fortunately, we found that we can detect similar peptides at a starting tissue volume of 0.0125 mm^3^, a magnitude of volume lower than our initial analysis. This is a feasible working volume and provides researchers with a starting point to plan and execute similar approaches to their studies.

An emerging theme is that the ECM is a driver of disease processes including atherosclerosis [52] fibrosis [22, 53, 54] and cancer [23]. The strategy developed here could be applied to a multitude of settings where tissue heterogeneity is a common theme. Currently, ECM tissue atlases of IPF [55] and, to an extent, liver fibrosis [56] have been developed to help researchers better understand and model fibrosis progression. Thus, this strategy could be applied to archived FFPE tissues to reliably determine not only regional ECM composition, but cellular pathways perturbed in health and disease.

The work described here could be enhanced by the combination of other ‘omic’ studies. For instance, serial sections following laser capture microdissection could be used for next-generation sequencing of RNA or DNA [57] for a more complete profiling of the regions of interest. In addition, matrix-assisted laser desorption/ionization (MALDI) could be applied to determine gradient changes at defined tissue interphases [58]. A limitation to this study is that H&E staining relies on pattern recognition rather than staining for specific proteins, however, our approach could be combined with spatially targeted optical microproteomics (STOMP) which combines antibodies and fluorescence to identify regions of interest [59].

## Conclusion

Our work is a step towards processing complex tissues even after formalin-fixation and hematoxylin and eosin-staining. The application of this novel microproteomics protocol, using commercially available tools, will enhance the development of comprehensive tissue atlases for a variety of pathologies.

## Supplementary information

**Additional file 1: Figure S1.** Protein extraction of H&E stained FFPE tissue sections. A 5-micron section of IPF tissue was serially sectioned and H&E stained. The whole tissue was used and subjected to 5% SDS alone, 5% SDS with heat-treatment, or 5% SDS treatment with heat-treatment followed by shearing in the presence of 7.5 M urea. Shown is a Sypro Ruby SDS-PAGE gel of complete lysates.

**Additional file 2: Table S1.** ECM constituents shared between morphologically normal alveoli and blood vessels.

**Additional file 3.**  BayesENproteomic analysis of proteins expressed between morphologically normal alveoli and blood vessels.

## Data Availability

Raw mass spectrometry data were deposited to ProteomeXchange with the identifier PXD014762.
