# Supplementary material for: Laser capture microdissection coupled mass spectrometry (LCM-MS) for spatially resolved analysis of formalin-fixed and stained human lung tissues
Source: Clin Proteomics. 2020 Jun 17;17:24. doi: 10.1186/s12014-020-09287-6 (PMC7302139; doi:10.1186/s12014-020-09287-6)
Supplement: Supplementary file 1 — Additional file 1: Figure S1. Protein extraction of H&E stained FFPE tissue sections. A 5-micron section of IPF tissue was serially sectioned and H&E stained. The whole tissue was used and subjected to 5% SDS alone, 5% SDS with heat-treatment, or 5% SDS treatment with heat-treatment followed by shearing in the presence of 7.5 M urea. Shown is a Sypro Ruby SDS-PAGE gel of complete lysates. [file 12014_2020_9287_MOESM1_ESM.docx]

**Supplemental Figure 1**


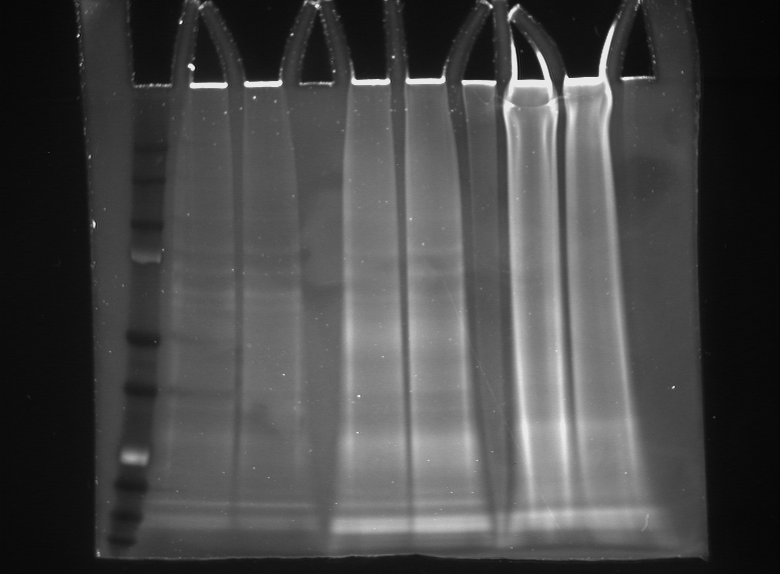


**7.5 M Urea + Shearing**

**Heat-retrieval**

**5% SDS**

**+**

**+**

**+**

**+**

**+**

**+**

**-**

**-**

**+**

**+**

**+**

**+**

**-**

**-**

**-**

**-**

**+**

**+**

**Supplemental Figure S1: Protein extraction of H&E stained FFPE tissue sections.** A 5-micron section of IPF tissue was serially sectioned and H&E stained. The whole tissue was used and subjected to 5% SDS alone, 5% SDS with heat-treatment, or 5% SDS treatment with heat-treatment followed by shearing in the presence of 7.5 M urea. Shown is a Sypro Ruby SDS-PAGE gel of complete lysates.
