# Supplementary material for: Laser capture microdissection coupled mass spectrometry (LCM-MS) for spatially resolved analysis of formalin-fixed and stained human lung tissues
Source: Clin Proteomics. 2020 Jun 17;17:24. doi: 10.1186/s12014-020-09287-6 (PMC7302139; doi:10.1186/s12014-020-09287-6)
Supplement: Supplementary file 2 — Additional file 2: Table S1. ECM constituents shared between morphologically normal alveoli and blood vessels. [file 12014_2020_9287_MOESM2_ESM.pdf]

Supplemental Table 1
(ECM constituents shared between morphologically normal alveoli and blood vessels)

UniProt	Protein name	Gene symbol	Accession
Q9Y6C2	EMILIN-1 (Elastin microfibril interface-located protein 1) (Elastin microfibril interfacer 1)	*EMILIN1*	EMIL1
P02452	Collagen alpha-1(I) chain (Alpha-1 type I collagen)	*COL1A1*	CO1A1
P02461	Collagen alpha-1(III) chain	*COL3A1*	CO3A1
P02462	Collagen alpha-1(IV) chain [Cleaved into: Arresten]	*COL4A1*	CO4A1
P05997	Collagen alpha-2(V) chain	*COL5A2*	CO5A2
P12110	Collagen alpha-2(VI) chain	*COL6A2*	CO6A2
P12111	Collagen alpha-3(VI) chain	*COL6A3*	CO6A3
P27658	Collagen alpha-1(VIII) chain (Endothelial collagen) [Cleaved into: Vastatin]	*COL8A1*	CO8A1
P12107	Collagen alpha-1(XI) chain	*COL11A1*	COBA1
Q99715	Collagen alpha-1(XII) chain	*COL12A1*	COCA1
P01040	Cystatin-A (Cystatin-AS) (Stefin-A) [Cleaved into: Cystatin-A, N-terminally processed]	*CSTA*	CYTA
P08311	Cathepsin G (CG) (EC 3.4.21.20)	*CTSG*	CATG
Q9UBR2	Cathepsin Z (EC 3.4.18.1) (Cathepsin P) (Cathepsin X)	*CTSZ*	CATZ
P07585	Decorin (Bone proteoglycan II) (PG-S2) (PG40)	*DCN*	PGS2
Q8IUX7	Adipocyte enhancer-binding protein 1 (AE-binding protein 1) (Aortic carboxypeptidase-like protein)	*AEBP1*	AEBP1
P30740	Leukocyte elastase inhibitor (LEI) (Monocyte/neutrophil elastase inhibitor) (EI) (M/NEI) (Peptidase inhibitor 2) (PI-2)	*SERPINB1*	ILEU
P23142	Fibulin-1 (FIBL-1)	*FBLN1*	FBLN1
P98095	Fibulin-2 (FIBL-2)	*FBLN2*	FBLN2
P35555	Fibrillin-1 [Cleaved into: Asprosin]	*FBN1*	FBN1
P02671	Fibrinogen alpha chain [Cleaved into: Fibrinopeptide A; Fibrinogen alpha chain]	*FGA*	FIBA
P02675	Fibrinogen beta chain [Cleaved into: Fibrinopeptide B; Fibrinogen beta chain]	*FGB*	FIBB
Q14112	Nidogen-2 (NID-2) (Osteonidogen)	*NID2*	NID2
Q63ZY3	KN motif and ankyrin repeat domain-containing protein 2 (Ankyrin repeat domain-containing protein 25) (Matrix-remodeling-associated protein 3)	*KANK2*	KANK2
P01023	Alpha-2-macroglobulin (Alpha-2-M) (C3 and PZP-like alpha-2-macroglobulin domain-containing protein 5)	*A2M*	A2MG
P04083	Annexin A1 (Annexin I) (Annexin-1) (Calpactin II) (Calpactin-2) (Chromobindin-9) (Lipocortin I)	*ANXA1*	ANXA1
P12429	Annexin A3 (35-alpha calcimedin) (Annexin III) (Annexin-3) (Inositol 1,2-cyclic phosphate 2-phosphohydrolase) (Lipocortin III)	*ANXA3*	ANXA3
P09525	Annexin A4 (35-beta calcimedin) (Annexin IV) (Annexin-4) (Carbohydrate-binding protein p33/p41) (Chromobindin-4) (Endonexin I)	*ANXA4*	ANXA4
P08758	Annexin A5 (Anchorin CII) (Annexin V) (Annexin-5) (Calphobindin I) (CBP-I) (Endonexin II) (Lipocortin V)	*ANXA5*	ANXA5
P02790	Hemopexin (Beta-1B-glycoprotein)	*HPX*	HEMO
P04196	Histidine-rich glycoprotein (Histidine-proline-rich glycoprotein) (HPRG)	*HRG*	HRG

Supplemental Table 1

(ECM constituents shared between morphologically normal alveoli and blood vessels)

O00468	Agrin [Cleaved into: Agrin N-terminal 110 kDa subunit; Agrin C-terminal 110 kDa subunit; Agrin C-terminal 90 kDa fragment (C90)	*AGRN*	AGRIN
Q86YZ3	Hornerin	*HRNR*	HORN
Q5D862	Filaggrin-2 (FLG-2) (Intermediate filament-associated and psoriasis-susceptibility protein) (Ifapsoriasin)	*FLG2*	FILA2
O15230	Laminin subunit alpha-5 (Laminin-10 subunit alpha) (Laminin-11 subunit alpha) (Laminin-15 subunit alpha)	*LAMA5*	LAMA5
P09382	Galectin-1 (Gal-1) (14 kDa laminin-binding protein) (HLBP14) (14 kDa lectin) (Beta-galactoside-binding lectin L-14-I) (Galaptin)	*LGALS1*	LEG1
Q14767	Latent-transforming growth factor beta-binding protein 2 (LTBP-2)	*LTBP2*	LTBP2
P51884	Lumican (Keratan sulfate proteoglycan lumican) (KSPG lumican)	*LUM*	LUM
P14543	Nidogen-1 (NID-1) (Entactin)	*NID1*	NID1
P20774	Mimecan (Osteoglycin) (Osteoinductive factor) (OIF)	*OGN*	MIME
P01009	Alpha-1-antitrypsin (Alpha-1 protease inhibitor) (Alpha-1-antiproteinase) (Serpin A1)	*SERPINA1*	A1AT
P35237	Serpin B6 (Cytoplasmic antiproteinase) (CAP) (Peptidase inhibitor 6) (PI-6) (Placental thrombin inhibitor)	*SERPINB6*	SPB6
P51888	Prolargin (Proline-arginine-rich end leucine-rich repeat protein)	*PRELP*	PRELP
P26447	Protein S100-A4 (Calvasculin) (Metastasin) (Placental calcium-binding protein) (Protein Mts1) (S100 calcium-binding protein A4)	*S100A4*	S10A4
P05109	Protein S100-A8 (Calgranulin-A) (Calprotectin L1L subunit) (Cystic fibrosis antigen) (CFAG) (Leukocyte L1 complex light chain)	*S100A8*	S10A8
P06702	Protein S100-A9 (Calgranulin-B) (Calprotectin L1H subunit) (Leukocyte L1 complex heavy chain)	*S100A9*	S10A9
P29508	Serpin B3 (Protein T4-A) (Squamous cell carcinoma antigen 1) (SCCA-1)	*SERPINB3*	SPB3
P21980	Protein-glutamine gamma-glutamyltransferase 2 (EC 2.3.2.13) (Tissue transglutaminase) (Transglutaminase C) (TG(C)) (TGC)	*TGM2*	TGM2
Q08188	Protein-glutamine gamma-glutamyltransferase E (EC 2.3.2.13) (Transglutaminase E) (TG(E)) (TGE) (TGase E) (Transglutaminase-3)	*TGM3*	TGM3
Q05707	Collagen alpha-1(XIV) chain (Undulin)	*COL14A1*	COEA1
P04004	Vitronectin (VN) (S-protein) (Serum-spreading factor) (V75) [Cleaved into: Vitronectin V65 subunit; Vitronectin V10 subunit]	*VTN*	VTNC
P04275	von Willebrand factor (vWF) [Cleaved into: von Willebrand antigen 2 (von Willebrand antigen II)]	*VWF*	VWF
Q96P63	Serpin B12	*SERPINB12*	SPB12
P55083	Microfibril-associated glycoprotein 4	*MFAP4*	MFAP4
P49257	Protein ERGIC-53 (ER-Golgi intermediate compartment 53 kDa protein) (Gp58) (Intracellular mannose-specific lectin MR60)	*LMAN1*	LMAN1
P55268	Laminin subunit beta-2 (Laminin B1s chain) (Laminin-11 subunit beta) (Laminin-14 subunit beta) (Laminin-15 subunit beta)	*LAMB2*	LAMB2

Supplemental Table 1

(ECM constituents shared between morphologically normal alveoli and blood vessels)

P02751	Fibronectin (FN) (Cold-insoluble globulin) (CIG) [Cleaved into: Anastellin; Ugl-Y1; Ugl-Y2; Ugl-Y3]	*FN1*	FINC
P50454	Serpin H1 (47 kDa heat shock protein) (Arsenic-transactivated protein 3) (AsTP3) (Cell proliferation-inducing gene 14 protein)	*SERPINH1*	SERPH
P17931	Galectin-3 (Gal-3) (35 kDa lectin) (Carbohydrate-binding protein 35) (CBP 35) (Galactose-specific lectin 3)	*LGALS3*	LEG3
Q9UBX5	Fibulin-5 (FIBL-5) (Developmental arteries and neural crest EGF-like protein) (Dance) (Urine p50 protein) (UP50)	*FBLN5*	FBLN5
P01011	Alpha-1-antichymotrypsin (ACT) (Cell growth-inhibiting gene 24/25 protein) (Serpin A3)	*SERPINA3*	AACT
P20908	Collagen alpha-1(V) chain	*COL5A1*	CO5A1
P01008	Antithrombin-III (ATIII) (Serpin C1)	*SERPINC1*	ANT3
P11047	Laminin subunit gamma-1 (Laminin B2 chain) (Laminin-1 subunit gamma) (Laminin-10 subunit gamma) (Laminin-11 subunit gamma)	*LAMC1*	LAMC1
P98160	Basement membrane-specific heparan sulfate proteoglycan core protein (HSPG) (Perlecan) (PLC)	*HSPG2*	PGBM
P13611	Versican core protein (Chondroitin sulfate proteoglycan core protein 2) (Chondroitin sulfate proteoglycan 2)	*VCAN*	CSPG2
P08572	Collagen alpha-2(IV) chain [Cleaved into: Canstatin]	*COL4A2*	CO4A2
P08123	Collagen alpha-2(I) chain (Alpha-2 type I collagen)	*COL1A2*	CO1A2
P12109	Collagen alpha-1(VI) chain	*COL6A1*	CO6A1
P08133	Annexin A6 (67 kDa calelectrin) (Annexin VI) (Annexin-6) (Calphobindin-II) (CPB-II) (Chromobindin-20) (Lipocortin VI)	*ANXA6*	ANXA6
P07339	Cathepsin D (EC 3.4.23.5) [Cleaved into: Cathepsin D light chain; Cathepsin D heavy chain]	*CTSD*	CATD
Q08380	Galectin-3-binding protein (Basement membrane autoantigen p105) (Lectin galactoside-binding soluble 3-binding protein)	*LGALS3BP*	LG3BP
Q15582	Transforming growth factor-beta-induced protein ig-h3 (Beta ig-h3) (Kerato-epithelin)	*TGFBI*	BGH3
P02679	Fibrinogen gamma chain	*FGG*	FIBG
P36955	Pigment epithelium-derived factor (PEDF) (Cell proliferation-inducing gene 35 protein) (EPC-1) (Serpin F1)	*SERPINF1*	PEDF
P19823	Inter-alpha-trypsin inhibitor heavy chain H2 (ITI heavy chain H2) (ITI-HC2) (Inter-alpha-inhibitor heavy chain 2)	*ITIH2*	ITIH2
P15502	Elastin (Tropoelastin)	*ELN*	ELN
P00488	Coagulation factor XIII A chain (Coagulation factor XIIIa) (EC 2.3.2.13) (Protein-glutamine gamma-glutamyltransferase A chain)	*F13A1*	F13A
P20073	Annexin A7 (Annexin VII) (Annexin-7) (Synexin)	*ANXA7*	ANXA7
P21810	Biglycan (Bone/cartilage proteoglycan I) (PG-S1)	*BGN*	PGS1
P00734	Prothrombin (EC 3.4.21.5) (Coagulation factor II)	*F2*	THRB
P19827	Inter-alpha-trypsin inhibitor heavy chain H1 (ITI heavy chain H1) (ITI-HC1) (Inter-alpha-inhibitor heavy chain 1)	*ITIH1*	ITIH1
Q9NVD7	Alpha-parvin (Actopaxin) (CH-ILKBP) (Calponin-like integrin-linked kinase-binding protein)	*PARVA*	PARVA
Q15063	Periostin (PN) (Osteoblast-specific factor 2) (OSF-2)	*POSTN*	POSTN
P39060	Collagen alpha-1(XVIII) chain [Cleaved into: Endostatin; Non-collagenous domain 1 (NC1)]	*COL18A1*	COIA1

Supplemental Table 1

(ECM constituents shared between morphologically normal alveoli and blood vessels)

P60903	Protein S100-A10 (Calpactin I light chain) (Calpactin-1 light chain) (Cellular ligand of annexin II)	*S100A10*	S10AA
P50995	Annexin A11 (56 kDa autoantigen) (Annexin XI) (Annexin-11) (Calcyclin-associated annexin 50) (CAP-50)	*ANXA11*	ANX11
P06703	Protein S100-A6 (Calcyclin) (Growth factor-inducible protein 2A9) (MLN 4) (Prolactin receptor-associated protein) (PRA)	*S100A6*	S10A6
P07355	Annexin A2 (Annexin II) (Annexin-2) (Calpactin I heavy chain) (Calpactin-1 heavy chain) (Chromobindin-8) (Lipocortin II)	*ANXA2*	ANXA2
